# Symptoms from Morphine

**Published:** 1897-03

**Authors:** W. H. Phillips

**Affiliations:** Germantown, Phila.


### SYMPTOMS FROM MORPHINE.

W. H. PHILLIPS, M. D., GERMANTOWN, PHILA.

The following symptoms were developed in a man who had accidentally taken an overdose of Morphine-sulphate, and whom I was summoned to see shortly after he had committed the fatal blunder.

They are offered as a suitable addition to the several contributions upon Morphine which have appeared in THE HOMŒOPATHIC PHYSICIAN from time to time.

The contributions referred to are the one by Dr. Rufus L.

---

**Vide* an article by R. H. Chittenden, Ph. D., *New York Medical Journal*, July 18th, 1896, p. 71.

Thurston in the December, 1895, number at page 563; that of Dr. Thomas Skinner, March, 1896, page 123; Dr. C. M. Boger's article, June, 1896, page 295, and Dr. E. V. Ross' addition in the number for December, 1896, page 524. These several contributions taken together with the following, form a very desirable addition to the pathogenesis of Sulphate of Morphia:

